# Superior Single-Entity Electrochemistry Performance of Capping Agent-Free Gold Nanoparticles Compared to Citrate-Capped Gold Nanoparticles

**DOI:** 10.3390/nano14171399

**Published:** 2024-08-28

**Authors:** Dain Heo, Ki Jun Kim, Seong Jung Kwon

**Affiliations:** Department of Chemistry, Konkuk University, 120 Neungdong-ro, Gwangjin-gu, Seoul 05029, Republic of Korea; hdi1405@konkuk.ac.kr (D.H.); kim5732@konkuk.ac.kr (K.J.K.)

**Keywords:** single-entity electrochemistry, gold nanoparticle, capping agent, hydrazine oxidation, glucose oxidation

## Abstract

In observing the electrocatalytic current of nanoparticles (NPs) using single-entity electrochemistry (SEE), the surface state of the NPs significantly influences the SEE signal. This study investigates the influence of capping agents on the electrocatalytic properties of gold (Au) NPs using SEE. Two inner-sphere reactions, hydrazine oxidation and glucose oxidation, were chosen to explore the SEE characteristics of Au NPs based on the capping agent presence. The results revealed that “capping agent-free” Au NPs exhibited signal magnitudes and frequencies consistent with theoretical expectations, indicating superior stability and catalytic performance in electrolyte solutions. In contrast, citrate-capped Au NPs showed signals varying depending on the applied potential, with larger magnitudes and lower frequencies than expected, likely due to an aggregation of NPs. This study suggests that capping agents play a crucial role in the catalytic performance and stability of Au NPs in SEE. By demonstrating that minimizing capping agent presence can enhance effectiveness in SEE, it provides insights into the future applications of NPs, particularly highlighting their potential as nanocatalysts in energy conversion reactions and environmental applications.

## 1. Introduction

In recent years, single-entity electrochemistry (SEE) has expanded beyond merely measuring nanoparticle (NP) sizes to include a wide range of studies on catalytic properties and reaction mechanisms [1,2,3,4,5,6,7]. SEE experiments typically involve NPs and ultramicroelectrodes (UMEs), employing detection strategies such as electrocatalytic amplification (EA) methods [8,9,10] and blocking strategies [11,12], depending on the catalytic activity of the NP materials. Materials like Pt and IrO_x_ are favored for EA methods due to their superior catalytic properties [8,13], while materials with lesser catalytic activity, such as Ag and polystyrene, are used in blocking strategies or self-oxidation reactions [14,15].

Gold (Au), known for its excellent catalytic properties, has been extensively synthesized and studied since Michael Faraday first synthesized Au colloids in 1857 [16]. Despite high expectations due to its versatile catalytic abilities, Au NPs have not been as widely used in SEE compared to Pt NPs [17]. Examples of Au NP applications in SEE include the following: The Unwin group demonstrated the effect of surface oxide formation on Au NPs [18]. The Long group showcased the intrinsic electrocatalytic activity of Au NPs [19]. The Jin He group illustrated the construction of plasmonic molecular junctions through Au NP collisions [20]. The Compton group introduced a sensing application of Au NPs using a capping agent [21,22].

Au NPs find extensive applications across various fields such as medicine [23,24], environment [25], energy [26], devices [27], electronics [28], sensors [29], etc. However, their utilization in SEE is limited. This limitation may be due to factors such as the reduced catalytic properties of NPs caused by stabilizing agents such as capping agents on the NP surface [30].

For catalytic reactions, the adsorption of reactants on the metal catalyst surface (in this case, Au) is crucial, especially for inner-sphere reactions [31]. However, during NP formation, the surface can be partially blocked by organic capping agents or polymers used to maintain colloidal stability. These auxiliary reagents passivate the NPs’ surface, and thus decrease their performance in applications like catalysis and surface-enhanced Raman scattering (SERs) [32].

As a result, NPs without capping agents are expected to potentially exhibit superior catalytic reactions [33,34,35]. However, due to issues such as the stability of synthesized NPs, there are few studies at the single-NP level examining these properties [16,17,18]. Recently, Kircher and colleagues reported a method for synthesizing Au NPs with minimal capping agent usage using H_2_O_2_ as a reducing agent through a seed-growth approach, referring to them as “capping agent-free” Au NPs [36].

The reduction potential of hydrogen peroxide can be easily adjusted by pH, and its molecular interactions with Au surfaces are not strong enough to cause passivation [37]. Therefore, the Au NPs synthesized by this method outperformed analogous NPs with capping agent and polymer coatings in both electrocatalysis and SERs.

In this study, we used two types of Au NPs with different capping agent concentrations to investigate the changes in SEE signals of Au NPs with varying capping agent levels for two inner-sphere reactions: hydrazine oxidation and glucose oxidation. We compared the SEE signals of commonly used citrate-capped Au NPs with those of “capping agent-free” Au NPs. By analyzing the magnitude and frequency of SEE signals, we evaluated and predicted the stability of Au NPs in electrolyte solutions based on the type of capping agent used. The information obtained from these observations regarding the correlation between capping agents and catalytic properties of nanoparticles can serve as crucial foundational knowledge for applying the catalytic properties of nanoparticles in various fields.

## 2. Materials and Methods

### 2.1. Reagent

Gold (III) chloride (HAuCl_4_, 30 wt. %), sodium borohydride (NaBH_4_, ≥96%), sodium citrate tribasic dihydrate, glucose, and all buffer salts were obtained from Sigma-Aldrich (St. Louis, MO, USA). Hydrogen peroxide (30.0~35.0 wt. %) was purchased from Samchun (Seoul, Republic of Korea). All chemicals were used as received. Ultrapure water (≥18 MΩ, Millipore, Burlington, MA, USA) was used in all experiments. Au wire was obtained from Goodfellow (Devon, PA, USA).

### 2.2. Preparation of Citrate-Capped Au NP

Citrate-capped Au NP was synthesized according to the procedure from a published paper [38]. First, 30 mL of 0.223 mM HAuCl_4_ solution was boiled in an oil bath with stirring and reflux. After boiling, 3 mL of 4.4 mM sodium citrate was dropwise to the boiling HAuCl_4_ solution. The mixture was boiled for 10 min more and then stirred for 15 min more at the room temperature. Then, a transparent reddish-pink Au NP solution can be obtained. The final concentration of citrate was 0.4 mM, because 3 mL of 4.4 mM citrate was added into the total solution volume of 33 mL. For calculating the concentration of the stock solution, the concentration of the Au precursor (0.2 mM) was divided by the average number of Au atoms in an average-sized Au NP. The calculated concentration of the stock solution was 500 pM. Considering the NP and citrate concentrations in the stock solution, approximately 8 × 10^5^ citrate molecules were associated with each single NP.

### 2.3. Preparation of “Capping Agent-Free” Au NP

The “capping agent-free” Au NP was synthesized by the seed-growth method [36]. Seed Au NP was synthesized in an ice bath. Briefly, 100 µL of 0.0254 M HAuCl_4_ solution, and 100 µL of 0.0254 M of sodium citrate solution were added into 9.8 mL of distilled water. Then, 300 µL of 0.1 M NaBH_4_ solution was dropwise to the mixture with vigorous stirring. The mixture was kept for 1 day for decomposing residual NaBH_4_. The concentration of citrate in the seed Au NP was 0.2466 mM, because 100 µL of 0.0254 M citrate was added into the total solution volume of 10.3 mL.

**Figure 1:**
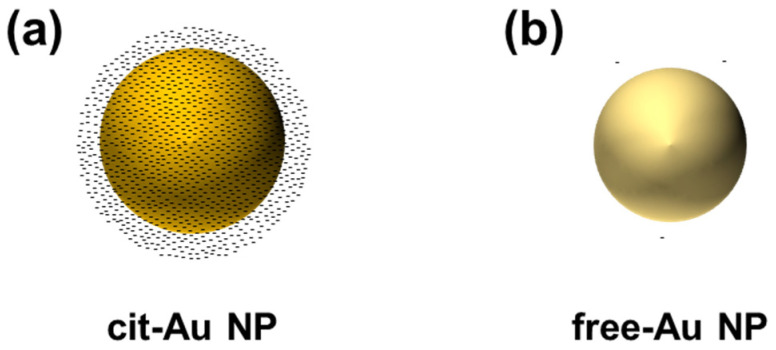
A schematic representation of the relative amount of citrate used in the synthesis of (**a**) a cit-Au NP and (**b**) free-Au NP.

To obtain spherical Au NPs, 50 µL of seed Au NP solution was added to 9.7 mL distilled water in a water bath (80 °C). Then, 60 µL of 0.0254 M HAuCl_4_ solution and 60 µL of 30 wt. % H_2_O_2_ were added into the diluted seed Au NP solution. This mixture was stirred for 40 min in a water bath (80 °C). The color of the mixture turned to transparent red. The final concentration of the citrate was 1.25 μM, because 50 μL of 0.2466 mM citrate was added into the total solution volume of 9.87 mL. And the final concentration of the Au precursor was 0.16 mM. Therefore, the concentration of the stock solution was calculated as 700 pM. Considering the NP and citrate concentrations in the stock solution, and assuming that all the citrate in the solution is present on the NP surface, approximately 1800 citrate molecules are associated with each single NP. This amount is 450 times smaller than that of citrate-capped Au NPs. The relative amounts of citrate present on the surface of the two types of Au NPs are schematically illustrated in Figure 1.

### 2.4. Preparation of UME

We followed a well-known process to prepare a Au (10 μm of diameter) UME [8,9]. Briefly, a Au wire was placed into a one-end-sealed capillary tube. The other side of the capillary tube was connected to a vacuum pump. Then, the end of a glass was heated by a nichrome coil under vacuum. After the Au wire was fixed to the tube, the capillary was polished with sandpaper to expose the Au wire and polished with alumina powder to obtain a mirror-like surface. A C-fiber UME (11 µm diameter) was obtained from BASi (West Lafayette, IN, USA).

### 2.5. Instrumentation

The electrochemical measurement was performed using a CHInstruments model 750e potentiostat (Austin, TX, USA) with the three-electrode cell system placed in a Faraday cage. A Pt wire and a Ag/AgCl (1 M KCl) electrode were used as the counter and the reference electrode, respectively. All potentials are reported vs. Ag/AgCl. Dynamic light scattering (DLS) and zeta potential analysis were conducted using a Zetasizer Nano ZS90 instrument (Malvern, Worcestershire, UK). For measuring DLS and zeta potential, we used DTS0012 and DTS1070, respectively. Transmission electron microscopy (TEM) and energy dispersive X-ray spectroscopy (EDS) measurements were conducted with JEM-F200 (JEOL, Tokyo, Japan). X-ray photoelectron spectroscopy (XPS) was conducted with Axis Supra+ (Kratos, Manchester, UK). The samples were drop-casted onto a glass (0.5 × 0.5 mm) and dried for 1 day in a vacuum oven. TEM, EDS, and XPS were conducted at the National Center for Inter-University Facilities (NCIRF, Seoul, Republic of Korea). Fourier-transform infrared spectroscopy (FTIR) was preformed using a Nicolet 6700 FT-IR spectrometer (Thermo Fisher Scientific, Waltham, MA, USA). The solution of the electrochemical cell contained 50 mM of a phosphate buffer (PB, pH 7) with 5 mM hydrazine for hydrazine oxidation and 0.1 M of NaOH with 30 mM glucose for glucose oxidation.

## 3. Results and Discussion

**Figure 2:**
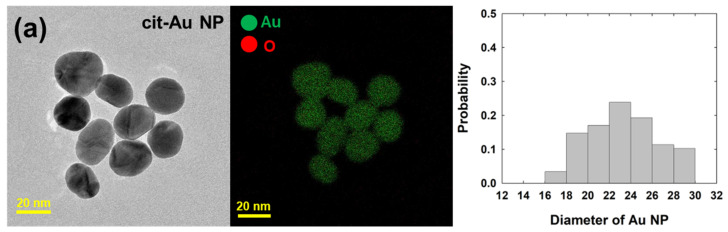

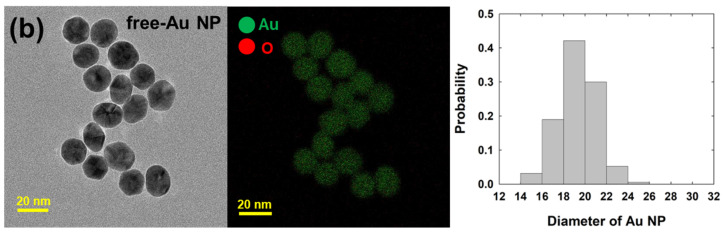
TEM and EDS measurement results and size distribution of (**a**) cit-Au NPs and (**b**) free-Au NPs. The average diameter of cit-Au NPs and free-Au NPs were 23.5 ± (3.5) and 19.5 ± (2.0) nm, respectively.

**Figure 3:**
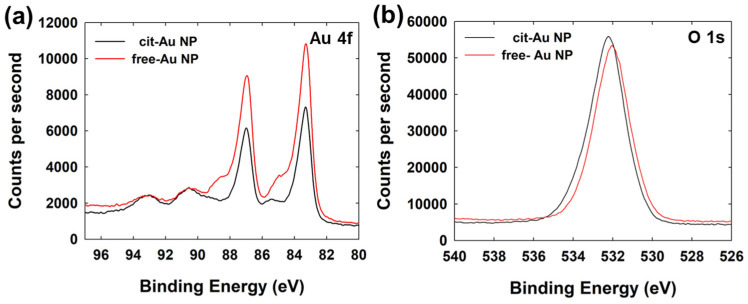
XPS spectra for (**a**) Au 4f and (**b**) O 1s for cit-Au NPs and free-Au NPs.

To characterize two synthesized Au nanoparticles (NPs), various analyses were conducted. Firstly, TEM measurements were performed to assess their size and surface topology. As shown in Figure 2, spherical Au NPs were observed. The diameters (±SD) of the citrate-capped Au NPs (cit-Au NPs) and the “capping agent-free” Au NPs (free-Au NPs) were 23.5 (±3.5) nm and 19.5 (±2.0) nm, respectively. As is generally known, it is difficult to detect the organic layer on the surface of nanoparticles using electron microscopy methods such as TEM. This is because the organic layer degrades due to the high-energy electron beam. To analyze the surface state of the NPs, energy dispersive X-ray spectroscopy (EDS) and X-ray photoelectron spectroscopy (XPS) measurements were carried out. As shown in Figure 2, EDS results indicated the presence of oxygen atoms on the surface in addition to Au, likely due to Au-oxide. As shown in Figure S1 (see the Supporting Information, SI), the atom % from EDS indicates a higher proportion of oxygen in free-Au NPs. XPS analysis was performed by coating the NPs onto an indium tin oxide (ITO) electrode. Figure 3 shows the peaks for Au 4f observed at 83 eV and 87 eV. The doublet peaks at 83 eV and 87 eV arise due to spin-orbit coupling caused by electron spin (Au 4f_7/2_ for 83 eV, and Au 4f_5/2_ for 87 eV) [38]. The free-Au NPs exhibited overall higher peak intensity compared to the cit-Au NPs.

Additionally, small shoulder peaks at 85 eV and 89 eV result from the increased binding energy caused by the electron density shift towards oxygen due to its higher electronegativity when Au is bonded to oxygen [39,40,41]. These shoulder peaks were observed more distinctly in the free-Au NPs compared to the cit-Au NPs. This is attributed to the Au-oxide on the surface being less shielded by capping agents like citrate in the free-Au NPs, allowing for greater exposure [36,42,43].

In the XPS analysis of O 1s, peaks were observed at 532.2 eV for cit-Au NPs and 531.9 eV for free-Au NPs. A survey spectrum of the XPS analysis is given in Figure S2.

The presence of a capping agent, citrate, was investigated via Fourier-transform infrared spectroscopy (FTIR). The C=O, C-O peaks are observed with different transmittance in the two Au NPs (Figure S3).

To measure the zeta potential of the NPs, dynamic light scattering (DLS) measurements were performed. As shown in Figure S4, the zeta potential values obtained were −31.8 mV for the cit-Au NPs and −23.8 mV for the free-Au NPs. In the case of free-Au NPs, it seems that a small amount of citrate added during seed formation, or anions from precursor reagents or buffer solutions, adsorbs on the surface to create a negative zeta potential and produce stable colloids [36,44].

Consequently, the results from the TEM, XPS, FTIR, and zeta potential analyses indicated that the two NPs synthesized via different methods (cit-Au NPs and free-Au NPs) have similar sizes of approximately 20 nm and possess stable colloidal states with nearly the same level of zeta potential. However, the larger Au peak and more pronounced Au-oxide peak observed in free-Au NPs suggest that their surface is more exposed, with fewer capping agents like citrate adsorbed on the surface.

To investigate the difference in the single-entity electrochemistry (SEE) signal according to the difference in capping agents between the two NPs, SEE experiments were conducted for two inner-sphere reactions: hydrazine oxidation and glucose oxidation.

**Figure 4:**
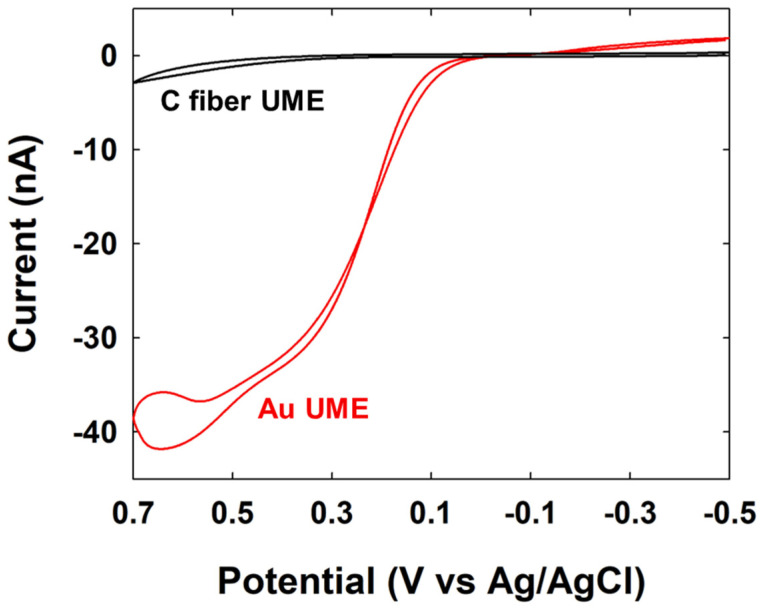
Cyclic voltammograms of C-fiber UME and Au UME in 5 mM hydrazine containing 50 mM PB (pH 7). Scan rate was 50 mV/s.

Firstly, to measure the SEE signal changes for hydrazine oxidation, a Au UME and C-fiber UME were prepared, and CV measurements were performed in a hydrazine solution. As shown in Figure 4, no electrocatalytic current for hydrazine oxidation was observed with the C-fiber UME, while an electrocatalytic current was observed starting at ~0.1 V (vs. Ag/AgCl) with the Au UME. Using the potential information of Au NP for hydrazine oxidation, Au NPs were injected into the electrolyte solution containing hydrazine, and the current signals of single-NP collisions on the UME were observed using the C-fiber UME.

**Figure 5:**
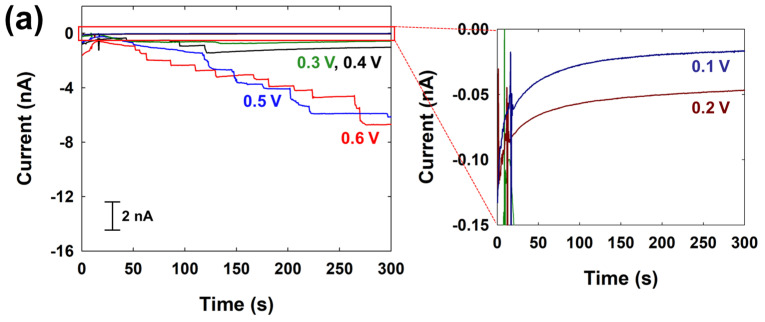

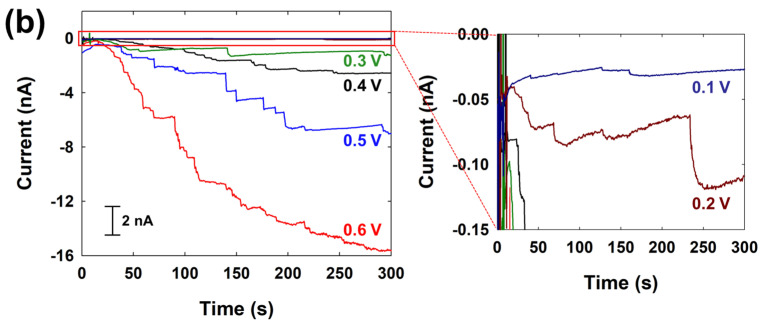
Chronoamperometric curves for the (**a**) cit-Au NPs (0.50 pM) and (**b**) free-Au NPs (0.7 pM) at different applied potentials from 0.1 to 0.6 V in 5 mM hydrazine containing 50 mM PB (pH 7). The data acquisition time was 50 ms.

SEE experiments were conducted with the cit-Au NPs and free-Au NPs on a C-fiber UME, applying potentials ranging from 0.1 V to 0.6 V. As shown in Figure 5, for cit-Au NPs, no SEE signals were observed at lower potentials such as 0.1 V and 0.2 V. SEE signals were only detected at a potential ≥ 0.3 V.

In contrast, for free-Au NPs, SEE signals were observed at ≥0.1 V, as theoretically expected, with a higher frequency of signals compared to cit-Au NPs at all tested potentials. The frequencies and magnitudes of SEE signals for hydrazine oxidation using both Au NPs are summarized in Table 1. As summarized in Table 1, the SEE signal frequency at 0.6 V was 0.13 (±0.02) s^−1^ pM^−1^ for cit-Au NPs and 0.31 (±0.02) s^−1^ pM^−1^ for free-Au NPs. The signal size tended to increase with the applied potential, as shown in Figure S5. At 0.6 V, the average signal magnitudes for cit-Au NPs and free-Au NPs were 246 (±181) pA and 161 (±88) pA, respectively. Cit-Au NPs are approximately 120% larger than free-Au NPs, but the SEE signal was about 150% greater.

In SEE experiments, the electrocatalytic current ($I_{SS}$) generated by a single NP can be calculated as follows [31]:

(1) $$I_{ss}=4\pi \left(ln2\right)nFDCr_{NP}$$

where n is the number of electrons transferred, *F* is the Faraday constant, *D* and *C* are the diffusion constant and concentration of the reactant (hydrazine or glucose), respectively, and $r_{NP}$ is the radius of the NPs.

Furthermore, when an NP collides and adsorbs onto an inert UME, the UME can be considered as a sink hole that captures the NP. The collision frequency (f), or signal frequency of the Au NPs, can be calculated using the following formula [45]:

(2) $$f=4D_{NP}C_{NP}r_{UME}$$

where $r_{UME}$ is radius of UME, and $D_{NP}$ and $C_{NP}$ are the diffusion coefficient and concentration of the Au NPs, respectively.

Based on these equations and considering the average size and size distribution of the NPs, the theoretically estimated collision frequency and average signal magnitude for hydrazine oxidation with cit-Au NPs and free-Au NPs are (0.33 (±0.03) s^−1^ pM^−1^, 141 (±15) pA) and (0.27 (±0.05) s^−1^ pM^−1^, 171 (±25) pA), respectively (Table 1).

In actual experimental results, we observe that the signal magnitude for cit-Au NPs increased while the frequency decreased. This trend is generally observed in SEE experiments because when NPs are injected into the electrolyte solution designed for catalytic reactions, changes in the pH or ionic strength of the solution can cause an aggregation of NPs. Specifically, an aggregation of citrate-capped Pt NPs in the presence of hydrazine has been previously reported [46]. Although Au NPs are used here instead of Pt NPs, the presence of citrate as a capping agent suggests the possibility of similar aggregation behavior. Therefore, the increase in average size due to the aggregation of cit-Au NPs likely leads to a higher signal magnitude and a corresponding decrease in frequency.

In contrast, the average signal magnitude and frequency for free-Au NPs align relatively better with the theoretical calculations. This suggests that free-Au NPs have a lower tendency to aggregate in the hydrazine solution, supporting their stability.

In summary, the experimental results suggest that cit-Au NPs exhibit aggregation in the presence of hydrazine, leading to larger signal magnitudes and lower collision frequencies. In contrast, free-Au NPs remain stable without significant aggregation, aligning well with theoretical predictions for both signal magnitude and frequency.

After performing single-NP collision experiments, the C-fiber UME would be decorated with Au NPs. Consequently, as shown in Figure S6, the Au NP-modified C-fiber UME exhibited catalytic characteristics similar to the Au UME rather than the C-fiber UME.

However, CV measurements revealed no significant catalytic current changes in the C-fiber UME coated with two different Au NPs. This indicates that the intrinsic catalytic properties of the two Au NPs are not significantly different in bulk analysis. Thus, at least in this experiment, the difference in SEE signals appears to be more sensitive to factors such as NPs’ aggregation and adsorption onto the electrode rather than the NPs’ unique catalytic properties.

**Figure 6:**
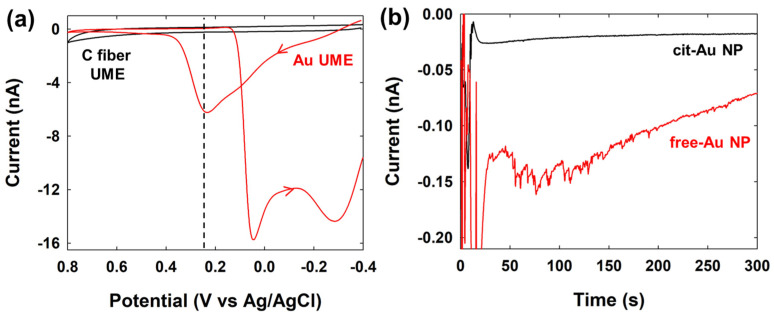
(**a**) Cyclic voltammograms of the C-fiber and Au UME in 0.1 M NaOH containing 30 mM glucose. The scan rate was 50 mV/s. (**b**) Chronoamperometric curves for the SEE of cit-Au NPs (1.0 pM) and free-Au NPs (1.4 pM) in 0.1 M NaOH containing 30 mM glucose. The applied potential was 0.25 V, and the data acquisition time was 50 ms.

To investigate the differences in SEE signals for another reaction, SEE experiments were conducted for glucose oxidation. Figure 6a depicts the electrocatalytic current responses of C-fiber UME and Au UME during glucose oxidation in a 0.1 M NaOH solution containing 30 mM glucose. The C-fiber UME showed no catalytic current, while the Au UME exhibited an electrocatalytic current peak around ~0.25 V. Since, as shown in Figure S7, SEE signals are significantly diminished >0.25 V due to the oxidation of Au, an applied potential of 0.25 V was chosen for the SEE of Au NPs on the C-fiber UME. The results of the SEE experiment, as shown in Figure 6b, reveal that cit-Au NPs exhibited almost no signal, whereas free-Au NPs showed very frequent signals [47]. The signal magnitudes and frequencies for glucose oxidation are also summarized in Table 1.

In this case, the absence of signals from cit-Au NPs is likely related to the stability of the NPs in the electrolyte solution (0.1 M NaOH) of high ionic strength. To investigate the stability of the NPs in different electrolytes, each electrolyte was prepared separately, and Au NPs were introduced to observe any change.

As shown in Figure S8, when DLS was measured after adding an excessive amount of Au NPs to each electrolyte, it can be seen that cit-Au NPs aggregate faster than free-Au NPs. In addition, in the case of cit-Au NPs, a large amount of cit-Au NP precipitation was observed after 1 day (Figure S9).

In conclusion, when observing SEE signals for cit-Au NPs and free-Au NPs in two catalytic reactions, free-Au NPs, which are more stable in the electrolyte solution of both reactions, exhibited signal magnitudes and frequencies consistent with theoretical values. In contrast, cit-Au NPs showed signals depending on the applied potential, and when observed, the signal magnitude was larger, and the frequency was smaller than theoretically expected. This discrepancy is likely due to the aggregation of NPs. When synthesizing free-Au NPs using the seed-growth method, H_2_O_2_ provides a finely tunable reduction potential range that controls the surface of the nanoparticles. This enables the synthesis of highly stable and high-performance NPs even in the absence of capping agents. As a result, aggregation is minimized, leading to clearer SEE signals compared to cit-Au NPs.

## 4. Conclusions

In conclusion, the catalytic properties of NPs are highly sensitive to the amount of capping agent present on their surface. Typical analytical methods, such as TEM and XPS, are challenging for detecting subtle changes in the amount of capping agent on the NP. However, the SEE method is an excellent tool for observing how variations in the amount of capping agent affect the catalytic properties of NPs. For the observation of electrocatalytic properties of Au NP at a single-NP level for hydrazine oxidation and glucose oxidation, capping agent-free Au NPs demonstrated significant advantages over citrate-capped Au NPs. The magnitude and frequency of the SEE signal of the capping agent-free Au NP match the theoretically calculated values. This means that the aggregation of the Au NP occurred only negligibly. Studies on the changes in catalytic properties of NPs due to capping agents can provide valuable insights into the synthesis and application of NPs. This suggests that SEE could be used as a method to study the electrocatalytic properties of NPs at the single-NP level by synthesizing NPs with various types and concentrations of capping agents in future research.

## Figures and Tables

**Table 1:** Signal magnitudes and frequencies of the SEE of free-Au NPs and cit-Au NPs.

		Free-Au NPs		Cit-Au NPs	
		Peak height (pA)	Frequency (s^−1^pM^−1^)	Peak height (pA)	Frequency (s^−1^pM^−1^)
Hydrazine Oxidation ^a^	Theoretical value	141 (±15)	0.33 (±0.03)	171 (±25)	0.27 (±0.05)
	Experimental value	161 (±88)	0.31 (±0.02)	246 (±181)	0.13 (±0.02)
Glucose Oxidation ^b^	Theoretical value	25 (±3)	0.33 (±0.03)	31 (±5)	0.27 (±0.05)
	Experimental value	6.4 (±5.2)	0.05 (±0.04)	-	-

^a^ Applied potential was 0.6 V. ^b^ Applied potential was 0.25 V.

## Data Availability

The raw data supporting the conclusions of this article will be made available by the authors on request.

## Supplementary Materials

The following supporting information can be downloaded at https://www.mdpi.com/article/10.3390/nano14171399/s1, Figure S1: EDS graphs and information about O-K and Au-M for (a) cit-Au NPs and (b) free-Au NPs. Figure S2: The survey spectrum of the XPS results for cit-Au NPs (black) and free-Au NPs (red). Figure S3: FTIR spectra for the cit-Au NPs and free-Au NPs. Figure S4: Zeta potential measurement of cit-Au NPs and free-Au NPs. Figure S5: SEE signal magnitude at a different applied potential. Figure S6: Cyclic voltammograms of C-fiber, cit-Au NP-decorated C-fiber, free-Au NP-decorated C-fiber, and Au UME in 5 mM hydrazine containing 50 mM PB (pH 7). Figure S7: SEE experiment for glucose oxidation at 0.3 and 0.35 V. Figure S8: DLS measurements for comparing the stability of each Au NP in electrolyte solutions. Figure S9: Photographs for comparing the stability of each NP in each electrochemical solution.
