# Supplementary material for: Superior Single-Entity Electrochemistry Performance of Capping Agent-Free Gold Nanoparticles Compared to Citrate-Capped Gold Nanoparticles
Source: Nanomaterials (Basel). 2024 Aug 28;14(17):1399. doi: 10.3390/nano14171399 (PMC11397711; doi:10.3390/nano14171399)
Supplement: Supplementary file 1 [file nanomaterials-14-01399-s001.zip › nanomaterials-3161222-supplementary.pdf]

Supporting Information

Superior Single-Entity Electrochemistry Performance of Capping Agent-Free Gold Nanoparticles Compared to Citrate-Capped Gold Nanoparticles

Dain Heo †, Ki Jun Kim † and Seong Jung Kwon *

Department of Chemistry, Konkuk University, 120 Neungdong-ro, Gwangjin-gu, Seoul 05029, Republic of Korea; hdi1405@konkuk.ac.kr (D.H.); kim5732@konkuk.ac.kr (K.J.K.)

* Correspondence: sjkwon@konkuk.ac.kr; Tel.: +82-2-450-0429

† These authors contributed equally to this work.

EDS measurements result

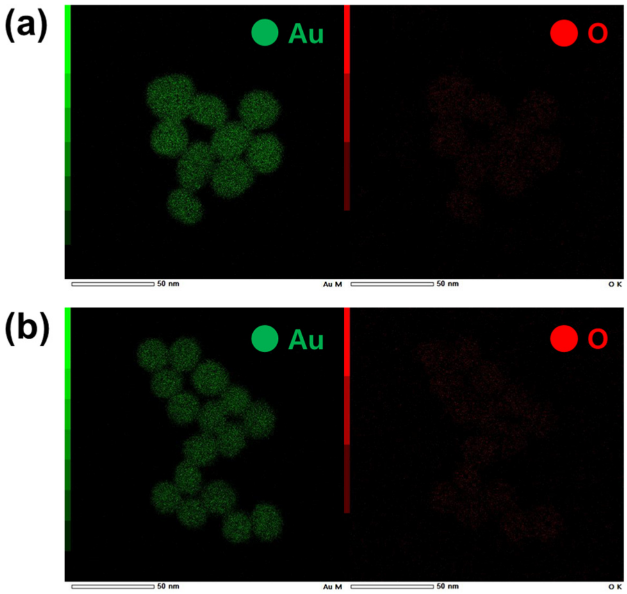

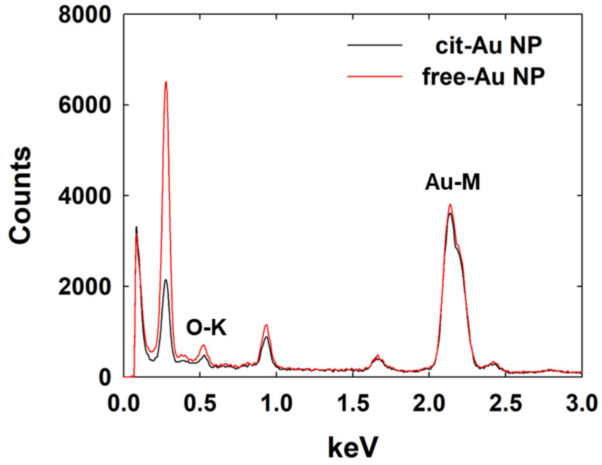

	Element	keV	Counts	Atom %
cit-Au NP	O-K	0.525	1340	17.2
	Au-M	2.120	33400	82.8
free-Au NP	O-K	0.525	2425	26.6
	Au-M	2.120	34600	73.4

Figure S1. EDS graphs and information about O-K, and Au-M for the (a) cit-Au NP and (b) free-Au NP .

The survey spectrum of the XPS result

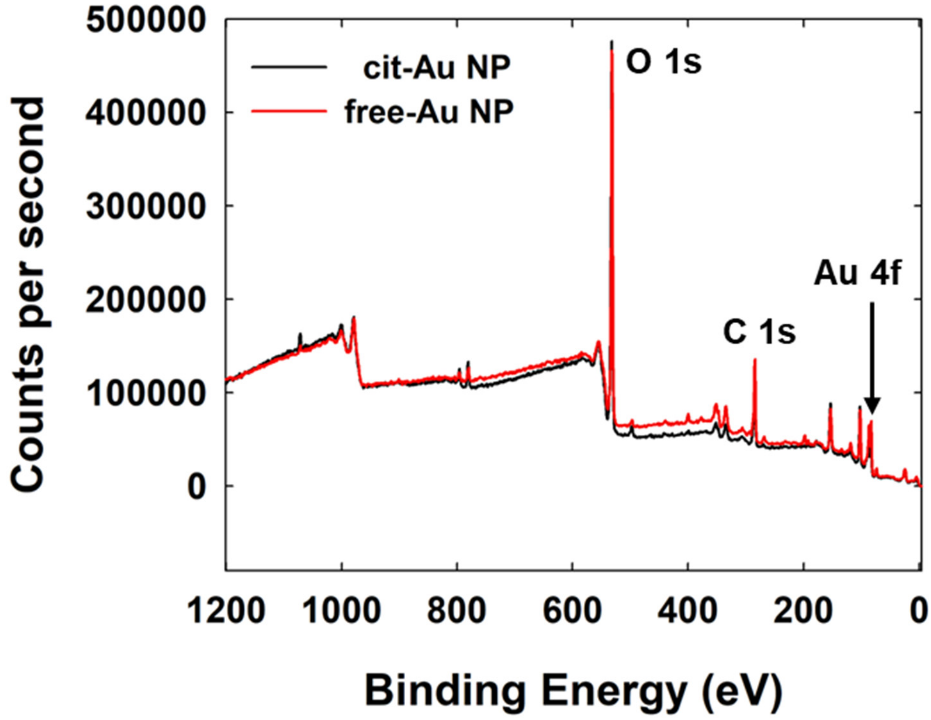

Figure S2. The survey spectrum of the XPS results for cit-Au NP (black) and free-Au NP (red).

FTIR measurements result

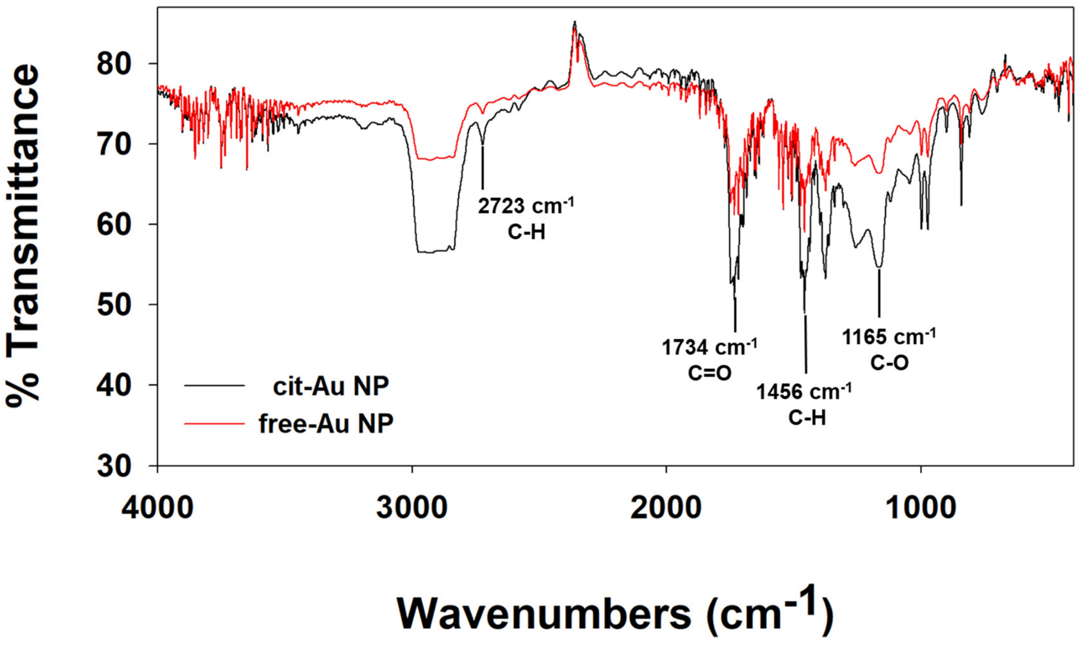

Figure S3. FTIR spectra for the cit-Au NP and free-Au NP.

Zeta potential measurements result

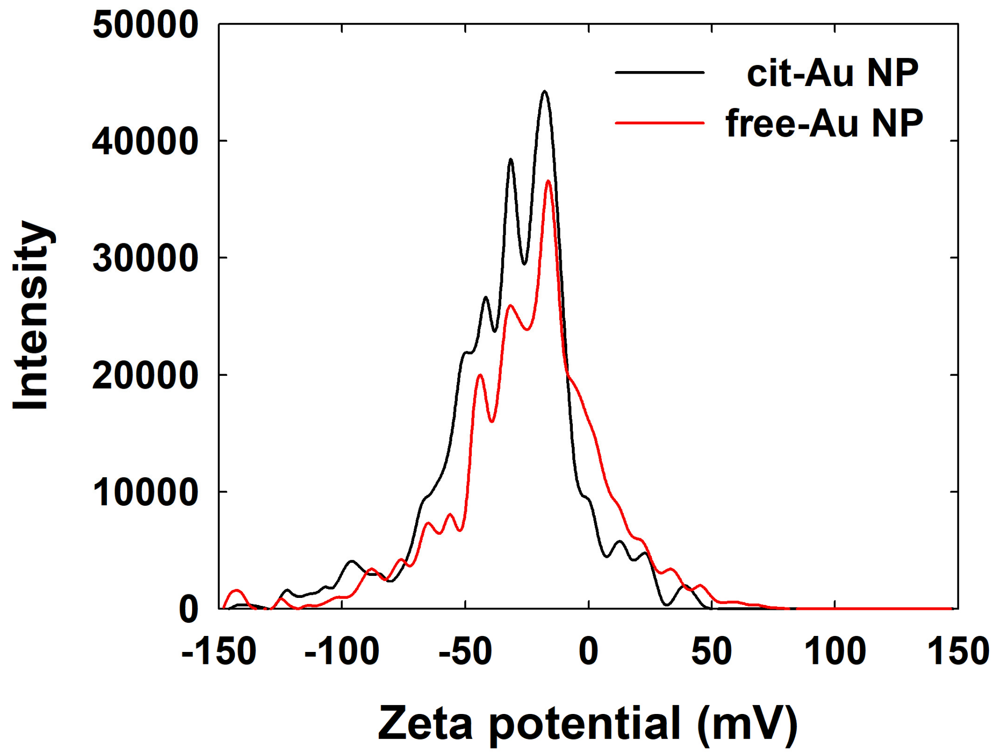

Figure S4. Zeta potential measurement of cit-Au NP and free-Au NP.

SEE signal magnitude at a different applied potential

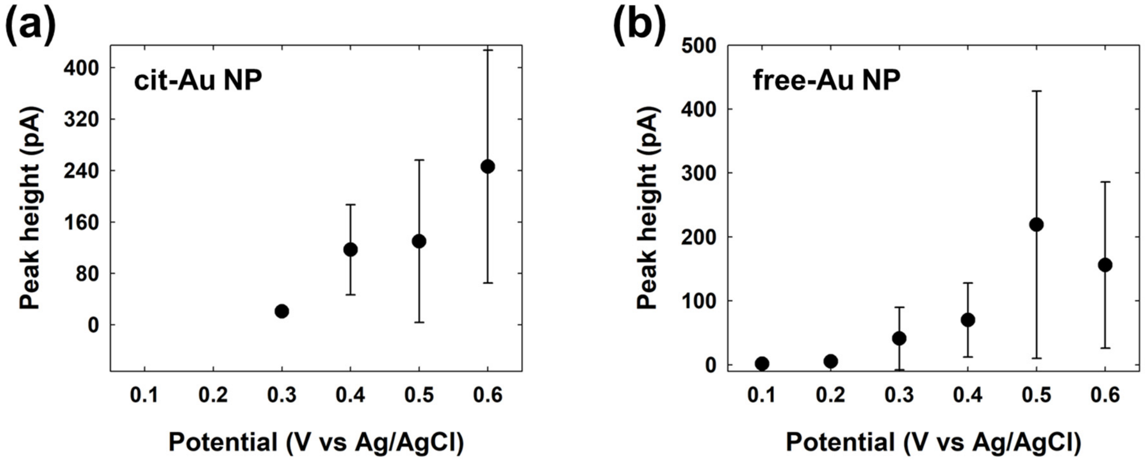

Figure S5. Average peak height for the (a) cit-Au NP (0.50 pM) or (b) free-Au NP (0.7 pM) at a different applied potential from 0.1 to 0.6 V in 5 mM hydrazine contained 50 mM PB (pH 7).

Cyclic voltammograms after SEE experiment with each Au NPs

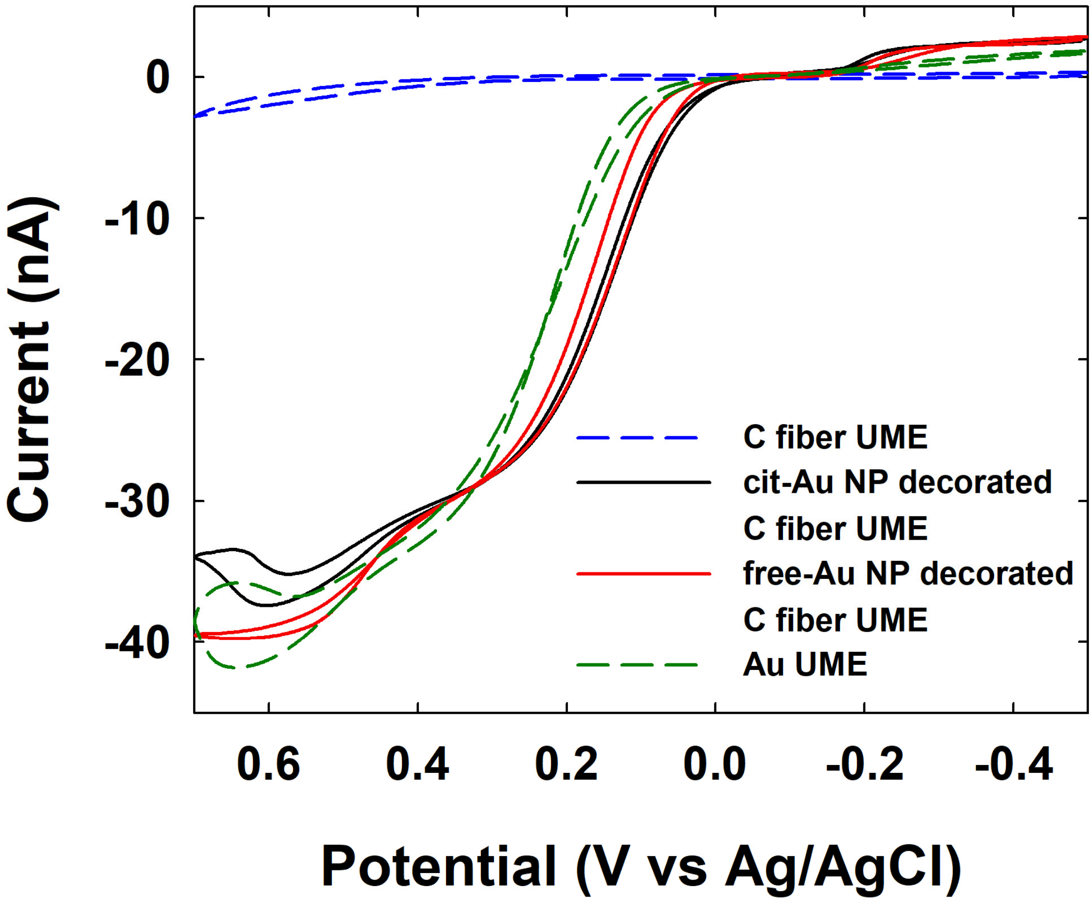

Figure S6. Cyclic voltammograms of C-fiber, cit-Au NP decorated C-fiber, free-Au NP decorated C-fiber, and Au UME in 5 mM hydrazine contained 50 mM PB (pH 7). Scan rate was 50 mV/s.

SEE experiment for glucose oxidation at 0.3 and 0.35 V

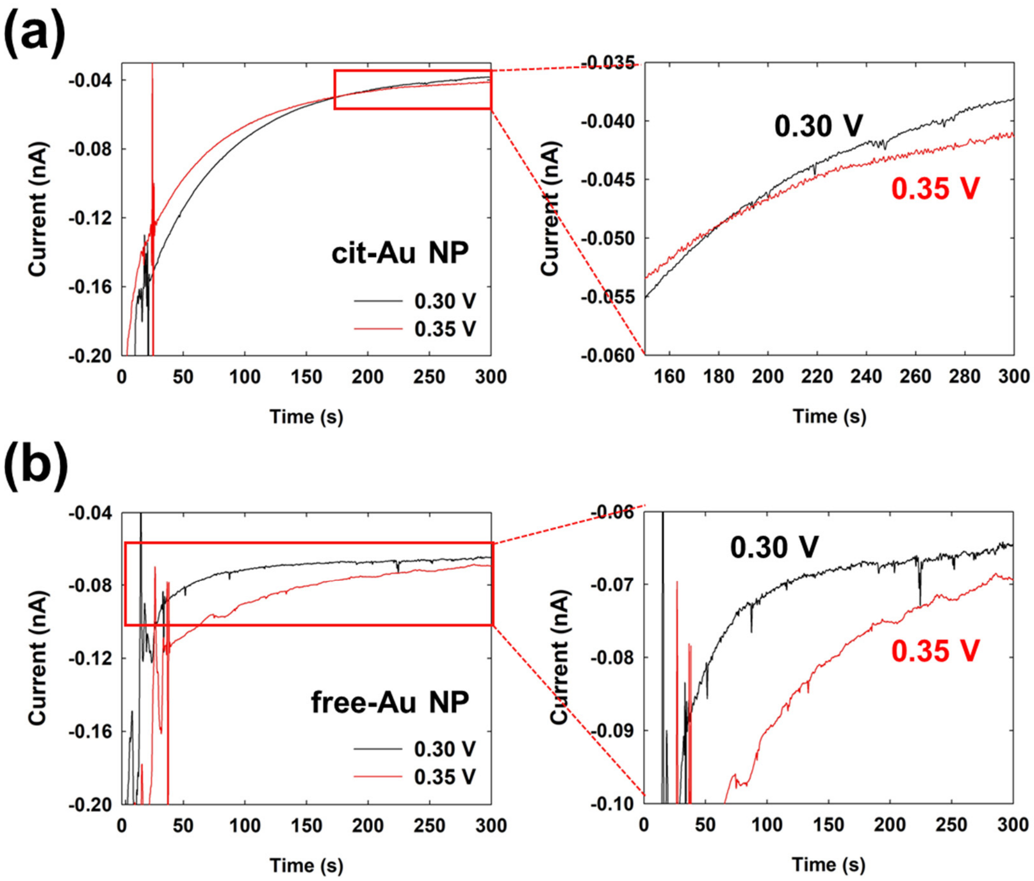

Figure S7. Chronoamperometric curves for the SEE of (a) cit-Au NP (1.0 pM) and (b) free-Au NP (1.4 pM) in 0.1 M NaOH containing 30 mM glucose. The applied potential was 0.30 and 0.35 V and the data acquisition time was 50 ms.

Size of each Au NPs in electrolyte solution measurements using DLS

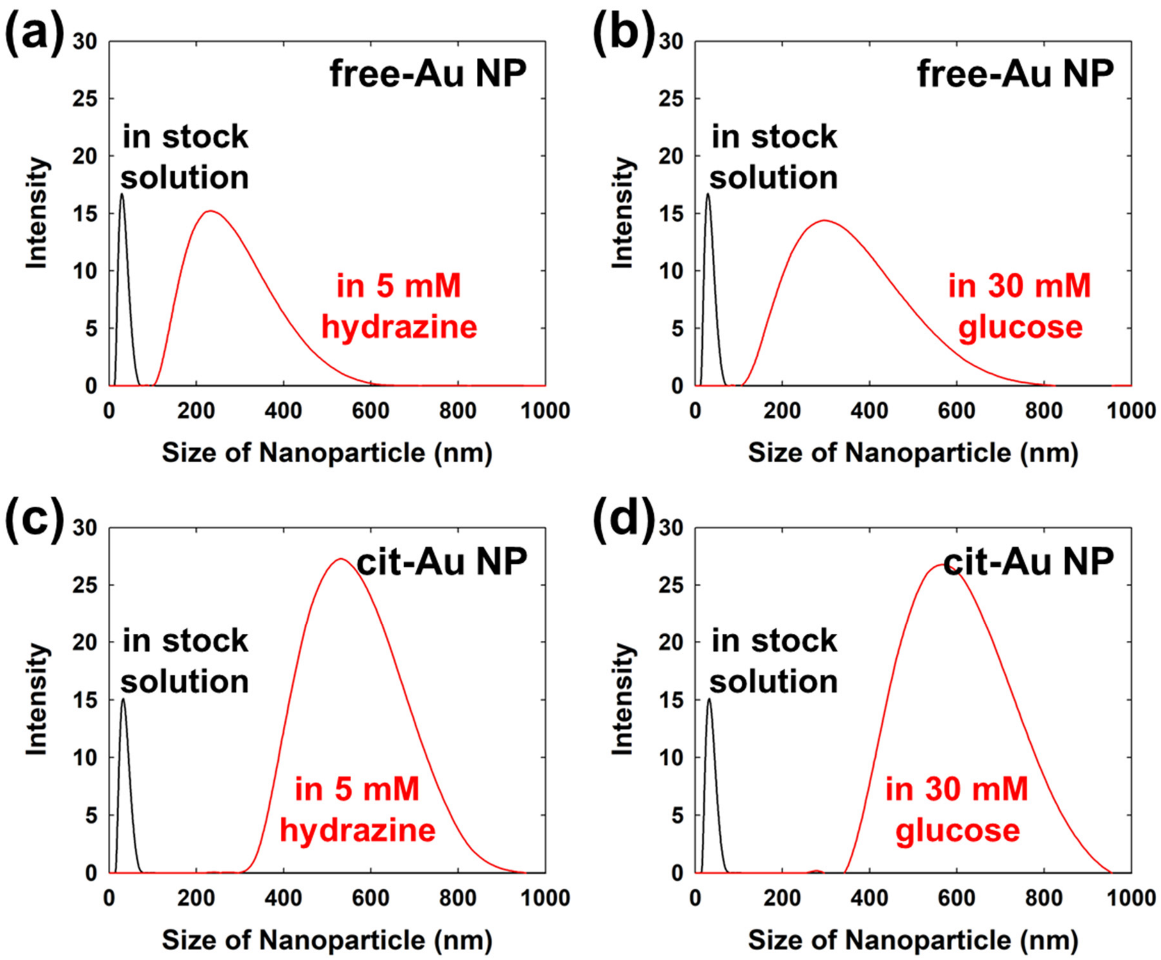

Figure S8. DLS measurements for comparing the stability of each Au NPs in electrolyte solutions. The free-Au NP in (a) 5 mM hydrazine containing 50 mM PB and (b) 30 mM glucose containing 0.1 M NaOH, respectively. The cit-Au NP in (c) 5 mM hydrazine containing 50 mM PB and (d) 30 mM glucose containing 0.1 M NaOH. Concentrations of free-Au NP, and cit-Au NP were 260 pM, and 190 pM, respectively.

Photographs of each Au NPs in electrolyte solution after 1 day

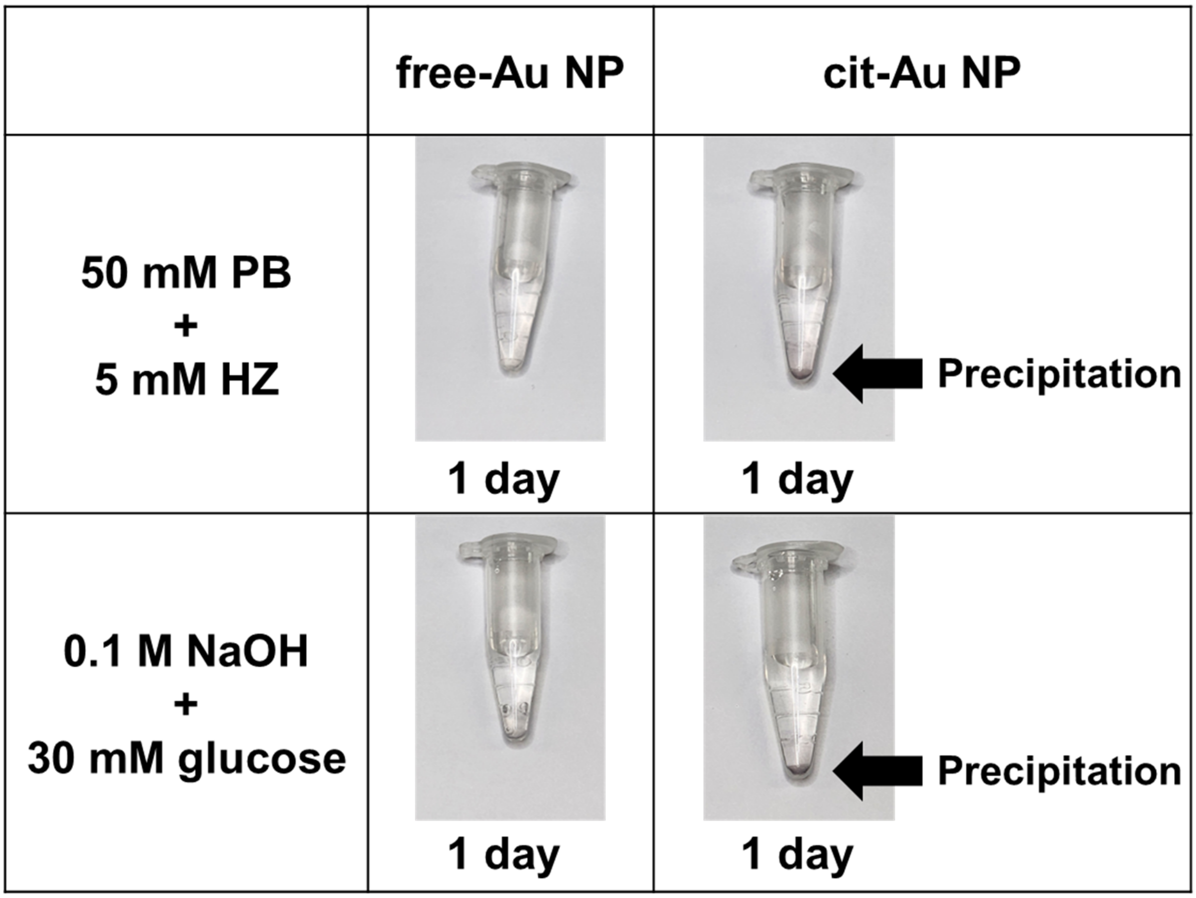

Figure S9. Photographs for comparing stability of each NPs in each electrochemical solutions. Concentrations of free-Au NP and cit-Au NP were 260 pM and 190 pM, respectively.
